# Diurnal Variations in Upper and Lower Body Power in Adolescent Volleyball Players: Exploring Time-of-Day Effects on Performance

**DOI:** 10.3390/sports12120320

**Published:** 2024-11-26

**Authors:** Nebojša Trajković, Vladan Milić, Tomislav Đurković, Tomica Rešetar, Georgiy Korobeynikov

**Affiliations:** 1 Faculty of Sport and Physical Education, University of Niš, 18000 Niš, Serbia; nele_trajce@yahoo.com; 2 Study Program of Sport and Physical Education, Department of Biomedical Sciences, State University of Novi Pazar, 36300 Novi Pazar, Serbia; vmilic@np.ac.rs; 3 Faculty of Kinesiology, University of Zagreb, 10000 Zagreb, Croatia; tomislav.djurkovic@kif.unizg.hr (T.Đ.); tomica.resetar@kif.unizg.hr (T.R.); 4 Department of Martial Arts and Power Sports, National University of Physical Education and Sport, 03150 Kyiv, Ukraine; 5 Department of Theory and Methodology of International Types of Wrestling, Uzbek State University of Physical Education and Sports, Tashkent 111700, Uzbekistan

**Keywords:** chronobiology, explosive strength, testing, young athletes

## Abstract

Background/Objectives: This study aims to investigate the daily variations in upper and lower body power performance in adolescent volleyball players. Methods: The sample consisted of 50 young male volleyball players (14.12 ± 0.8 years), actively involved in regular training and competition. Players were tested for vertical jump tests and medicine ball throws twice, once in the morning (8:00–9:30 h) and once in the evening (18:00–19:30 h). Results: Significant differences (*p* < 0.05; ES = 0.35–0.42) in vertical jump were observed when comparing the morning and evening performance except for counter movement jump with arm swing, where there were no significant differences (*p* = 0.21). The results for the upper body power tests revealed a significant difference only in the standing medicine ball throw (*p* = 0.05; ES = 0.35). There were no significant differences in lying and seated medicine ball throw (*p* > 0.05). Conclusions: This study demonstrated that lower body power, manifested in vertical jump performance, was significantly better in the evening compared to the morning. For upper body assessments, the standing medicine ball throw appears more reflective of volleyball-specific movements, while the lying and sitting throw may be less applicable. These findings suggest that volleyball training and testing, especially for leg power, may be more effective later in the day, while upper body performance appears less affected by time.

## 1. Introduction

Volleyball performance in younger players is heavily reliant on developing core skills like agility and coordination. As players are still maturing physically, their performance can vary significantly based on growth patterns and motor skill development, making training and assessment particularly important during this stage [1]. Later during children’s growth, strength performance becomes one of the crucial factors for young athletes [2], particularly in sports like volleyball, where explosive movements and power generation are essential. Power is one of critical components of performance during adolescence, contributing to better jumping, sprinting, and change of direction speed [1]. Malina et al. [2] further emphasize that variations in maturity status can contribute to differences in power performance during this developmental stage.

In recent years, there has been increasing interest in understanding the role of daily variations, or circadian rhythms, in physical performance [3,4,5,6,7,8]. Circadian rhythms are internal biological processes that follow a roughly 24-h cycle, significantly impacting various physiological factors, including core body temperature, hormone levels, and neuromuscular function, all of which contribute to fluctuations in athletic performance throughout the day [9]. Research indicates that core body temperature, a key component influenced by circadian rhythms, generally peaks in the late afternoon, which aligns with increased muscle flexibility, enzyme activity, and reaction times, thus enhancing physical performance in activities requiring strength and power [10,11]. Additionally, hormonal fluctuations, such as cortisol and testosterone levels, vary by time of day, with testosterone, associated with muscle anabolism, often higher in the morning, and cortisol, a stress-related hormone, decreasing throughout the day. These hormonal rhythms play a complex role in energy availability and muscle recovery [12,13]. Consequently, these circadian-regulated physiological variations suggest that testing and training scheduled at different times of day may yield significantly varied performance outcomes, which is crucial for optimizing training programs and accurate athlete assessment [5,14]. This phenomenon can be particularly relevant for young athletes, as their physiological responses to training may differ based on time of day.

Earlier studies, conducted in basketball, handball, and soccer, have shown that the time of day significantly affects athletic performance, including strength and short-term maximal efforts. For example, Chtourou and Souissi [10] examined the effects of training at specific times of the day, finding that afternoon sessions typically result in better strength performance than morning ones. Mhenni et al. [15] demonstrated that team-sport athletes, such as female handball players, performed better in short-term maximal efforts during the evening. Similarly, Pavlović et al. [6] examined elite male handball players and found significant differences in performance between morning and evening sessions. Roveda et al. [8] explored the influence of chronotype on motor skills in adolescent soccer players, revealing that athletes’ performance can vary depending on their internal biological clocks. The study found that motor skills, such as coordination and speed, were significantly impacted by whether players were morning or evening types. These findings are relevant to volleyball as well, where athletes may experience similar fluctuations in strength and power output throughout the day. Understanding these variations is crucial to avoid misjudging an athlete’s capabilities and to optimize both testing and training sessions accordingly.

Most studies in volleyball showed that physical performance variability is influenced by circadian rhythms and chronotype preferences. Pivovarnicek et al. [7] explored Slovak men’s volleyball players, finding that circadian preference affects performance based on the time of day athletes train. Zhu and Cui [16] highlighted the importance of aligning plyometric jump training with athletes’ chronobiological rhythms to optimize adaptation. Similarly, Martín-López et al. [17] reported that neuromuscular performance in female volleyball players varies depending on time-of-day and chronotype, suggesting a need for individualized training schedules.

For young volleyball players, strength and conditioning play a vital role in enhancing performance, preventing injuries, and improving overall athletic development [18]. However, the timing of strength training and its impact on performance is often overlooked. Exploring how strength performance varies throughout the day can provide valuable insights for optimizing training programs and maximizing performance outcomes [10]. Daily variations in performance hold significant importance when it comes to testing and evaluating volleyball players [17]. Testing at different times of the day could result in varying outcomes, which might not accurately reflect an athlete’s true potential [6]. For instance, an athlete tested in the morning might exhibit lower strength levels compared to an afternoon session, due to circadian influences [19]. This was confirmed by Knaier et al. [20], who showed that maximum strength performance typically peaks in the afternoon or evening, while morning performance can be hindered due to lower body temperature and hormonal concentrations. Understanding the timing of peak performance is essential for optimizing training and competition schedules for adolescents, as it helps align physical efforts with periods of peak physiological readiness [21]. Thus, understanding these variations is essential to ensure precise assessments and better performance analysis.

This study aims to investigate the daily variations in upper and lower body power performance in young volleyball players, exploring how circadian rhythms influence performance throughout the day. By examining how strength levels change throughout the day, this research seeks to provide a clearer understanding of optimal training times and to contribute to the growing body of literature on circadian influences in sports. The findings could have significant implications for developing tailored training regimens that align with an athlete’s natural performance rhythms. It was hypothesized that both lower and upper body strength would be better in the evening compared to morning.

## 2. Materials and Methods

### 2.1. Participants

The sample consisted of 50 male volleyball players (age = 14.5 ± 0.5 years; Y-PHV, i.e., years to and from peak height velocity = 0.6 ± 0.9 years), actively involved in regular training and competition (Table 1). These participants were selected based on the following criteria: they were active members of volleyball clubs in Serbia, had been training for at least four years, and participated in regular training sessions scheduled three times a week, each lasting 80–90 min. All participants were actively competing in the regional league and were members of teams that ranked within the top eight in their age group at the national level. This ensures that the sample consisted of well-trained, competitive athletes with a consistent background in volleyball. All participants were required to be healthy, free from recent or current injuries, and had undergone medical examinations. For each participant, maturity was estimated by predicting age at peak height velocity (Y-PHV) [2] via a gender-specific equation that includes anthropometric measures (weight, height, leg length, and sitting height) [22], as this has been considered as the directive for assessing physical fitness in boys and girls aged 10–16 [23]. Parental consent was obtained for participation in the experiment and testing. The study was conducted by an experienced team of researchers, and all procedures were approved by the Faculty of Sport and Physical Education, University of Novi Sad, Ethical board (Ref. No. 27/2019).

### 2.2. Procedures

The testing procedure was conducted indoors in a standard volleyball hall under controlled environmental conditions to ensure consistency and accuracy. The temperature inside the hall was maintained between 20–22 °C, with no wind or external weather influences, providing an optimal setting for performance. The testing surface was a non-slip hardwood floor, and proper lighting was ensured throughout the hall. Participants wore their regular sports attire, including athletic shoes. Participants underwent a familiarization session prior to testing, which included detailed instructions, demonstrations, and practice trials for both the vertical jump and medicine ball throw tests. This session was conducted to ensure consistent technique and reduce potential learning effects during actual testing. Before testing, all participants completed a standardized warm-up consisting of dynamic stretching and light jumping exercises to prepare for maximal effort during the explosive power tests. The testing procedures were conducted twice—once in the morning (8:00–9:30 h) and once in the evening (18:00–19:30 h)—with the same participants and the same researchers to ensure consistency. The timing of the morning and evening testing sessions was consistent with those used in earlier studies [6,10,15]. The selected testing times align with known circadian peaks and troughs in physiological function [20]. These timeframes provide a clear contrast for examining diurnal variations in performance, particularly in adolescent athletes whose circadian rhythms may be undergoing developmental shifts. Testing was conducted in the same indoor sports hall for both morning and evening sessions, ensuring controlled and stable conditions. The temperature in the hall was maintained at approximately 20–22 °C, with similar lighting and noise levels for both time periods, minimizing external factors that could influence performance outcomes. The same researchers supervised both sessions to eliminate variations in measurement or instruction, ensuring reliability and comparability between the morning and evening tests. To address potential confounding factors, we instructed participants to maintain their usual dietary habits throughout the testing day, specifically advising them to avoid caffeine and any supplements that could affect their circadian rhythm. Participants were reminded to stay hydrated and to follow similar sleep schedules the night before the testing session. Additionally, participants were advised to refrain from high-intensity physical activities for at least 48 h before testing. By standardizing these conditions, we aimed to minimize external influences and create a controlled environment that levels both morning and evening testing conditions across participants.

### 2.3. Testing

The testing began with the measurement of participants’ anthropometric characteristics, including height and body mass. Body height was measured using an anthropometer (SECA 214, Hamburg, Germany) following standard procedures, with the subject standing upright in the Frankfurt plane position. Body mass was recorded using the Omron BF511 scale (Kyoto, Japan). Body mass index (BMI) was calculated using the formula BMI = weight/height^2^. Following these baseline measurements, participants proceeded to explosive strength tests. Each participant performed three trials for both the vertical jump and medicine ball throw tests, with standardized rest intervals of 2 min between trials to prevent fatigue. Additionally, all participants were given consistent instructions and cues to ensure standardized testing conditions across sessions.

### 2.4. Vertical Jump Tests

The procedures for the vertical jump tests were performed using the OptoJump system (Microgate, Bolzano, Italy), which provides precise measurements of jump height and power. Three types of jumps were tested: squat jump (SJ), countermovement jump (CMJ), and countermovement jump with arm swing (CMJAS).

During the SJ test, the athlete stands upright within the Optojump measuring cells with both feet on the ground. Starting from a static squat position (approximately 90 degrees knee bend), the athlete holds still for a few seconds to eliminate countermovement. Then, they explosively extend their legs to jump vertically as high as possible, with no swinging of the arms, as hands remain on the hips throughout the movement. Jump height is measured in centimeters using the Optojump system.

In the CMJ test, the athlete also stands upright within the Optojump system with feet shoulder-width apart. They initiate the movement by quickly dropping into a partial squat (eccentric phase), followed by an immediate and explosive upward jump (concentric phase). Arms remain on the hips during this test as well, preventing arm swing from contributing to the jump. The jump height is recorded using the Optojump system in centimeters.

For the CMJ with Arm Swing, the athlete begins in the same upright position, but this time they are allowed to use their arms for additional momentum. The movement begins with a downward squat (eccentric phase) while simultaneously swinging the arms back. As the athlete jumps, they swing their arms forward and upward to generate additional force and height. This added arm motion typically results in a higher jump compared to the SJ and CMJ. Jump height is again measured in centimeters by the Optojump system.

In all tests, athletes perform three attempts, and the highest recorded jump from each test is used for further analysis [24]. This ensures that the peak performance is captured for each jump type, allowing for more accurate assessment of lower body power. The order of different types of jump was assigned randomly for each participant. The reliability for all tests was confirmed previously [25]. Results showed excellent reliability (interclass correlation coefficients ≥ 0.97) for all vertical jumps in professional volleyball players.

### 2.5. Block and Attack Jump

For specific volleyball jump tests, we have used previously explained reliable and validated tests, Block and Attack jump [24,25,26,27]. The reliability of the jumping tests ranged from 0.97 to 0.99 for interclass correlation coefficients. In these specific volleyball tests for explosive vertical jump power, participants first stood next to a wall to measure their standing reach height by extending one arm and marking the height on the wall. The measurement of the standing reach height is used for a calculation of the relative jump height on the jumping tasks i.e., absolute jump height – standing reach height = relative jump height [28]. After recording this, participants positioned themselves beneath a Vertec device. They performed a countermovement jump, aiming to touch and displace the highest possible vanes on the Vertec. For the attack jump test, the subject’s reach height was first measured while standing in front of the wall and by extending one arm and marking the height on the wall. The jump begins with a three-step approach, where the subject accelerates toward the target before jumping with both legs to simulate a volleyball spike. The reach height after the jump is recorded, and the difference between the initial reach and the jump is used to calculate jump height. For the block jump, the participant starts facing the wall marking the standing height on the wall. The participants then stand in front of Vertec device with arms positioned in front as if preparing to block. The subject performs a countermovement and jumps vertically to simulate a blocking action, swinging the arms upward during the jump to reach as high as possible. The initial reach height from a standing position is subtracted from the block jump height to determine the final jump performance. Three attempts are recorded for both tests, and the best jump height is used for further analysis [24]. Participants wore athletic shoes during testing.

### 2.6. Upper Body Explosive Strength

The procedures for testing explosive strength of the upper body were conducted using three different medicine ball throw tests: Laying medicine ball (MB) throw, Seated MB throw, and Standing MB throw. Participants performed a 10-min warm-up that consisted of exercises used to warm up the shoulders plus submaximal throwing efforts with lighter balls (3–5 throws with 1–2 kg weighted ball). Before the test, the participants performed familiarization with the process of throwing using the light weighted balls and all throwing techniques. Reliability coefficient (ICC) for all MB tests was r = 0.87–0.96.

Lying MB Throw from Supine Position: The original test was previously introduced with lighter medicine ball [29]. In the current study, a modified test was used with heavier medicine balls previously validated in earlier study [30]. The participant lay on a mat with legs slightly apart and extended toward the measuring scale. Holding a 3 kg medicine ball with both hands, the participant extended the arms fully before performing a maximal throw towards the scale without lifting the head from the mat. The result is the distance in meters. Three throws were performed, with the best throw used for analysis. The ball was returned to the participant between attempts.

Standing MB Forward Throw: The test was previously used and validated in volleyball [31]. Participants stood behind the starting line, feet a shoulder-width apart, holding a 3 kg medicine ball behind their head. No preliminary steps were allowed before the throw [32]. They threw the ball forward as far as possible, with the heels allowed to lift but the toes remaining on the ground. No forward movement was allowed after the throw. Each participant completed three throws, and the best distance in meters was recorded.

Seated MB Chest Throw from Seated Position was modified from original test performed by Van Den Tillaar and Marques [32]. Participants sat on a bench with adjustable back support, thighs horizontal, knees bent at 90°, and ankles secured by rotating pads. Strapped in with an elastic band around the chest, participants held the 3 kg medicine ball at chest level and performed an explosive push forward at an approximate 30° angle. The surface of the ball was lightly wetted to mark the landing point, and three throws were performed with a 2 min rest between each. The best throw was marked in meters and used for analysis.

Each test utilized a 3 kg medicine ball (Tigar MB3, 21.5 cm diameter) for consistency.

### 2.7. Statistical Analysis

The required sample size for the study was calculated with G power using a within-subject design, with a medium effect size (Cohen’s d = 0.5), a significance level of 0.05, and a desired power of 0.80, resulting in a minimum of 34 participants. With 50 participants, the study is sufficiently powered (0.93) to detect significant differences in performance. All data are presented as means ± standard deviation. Since normality of data distribution was confirmed by the Kolmogorov–Smirnov test, any systematic daily changes in physical fitness testing performance were assessed using paired sample T test. Additionally, effect size [33] was evaluated using Cohen’s d (standardized mean differences with a 95% confidence interval). Based on Cohen’s guidelines, the effect size is considered trivial (<0.2), small (0.2–0.5), medium (0.5–0.8), or large (>0.8). All statistical analyses were performed using SPSS 24.0 software (SPSS Inc., Chicago, IL, USA). The alpha level was set at <0.05 to indicate statistical significance.

## 3. Results

**Figure 1:**
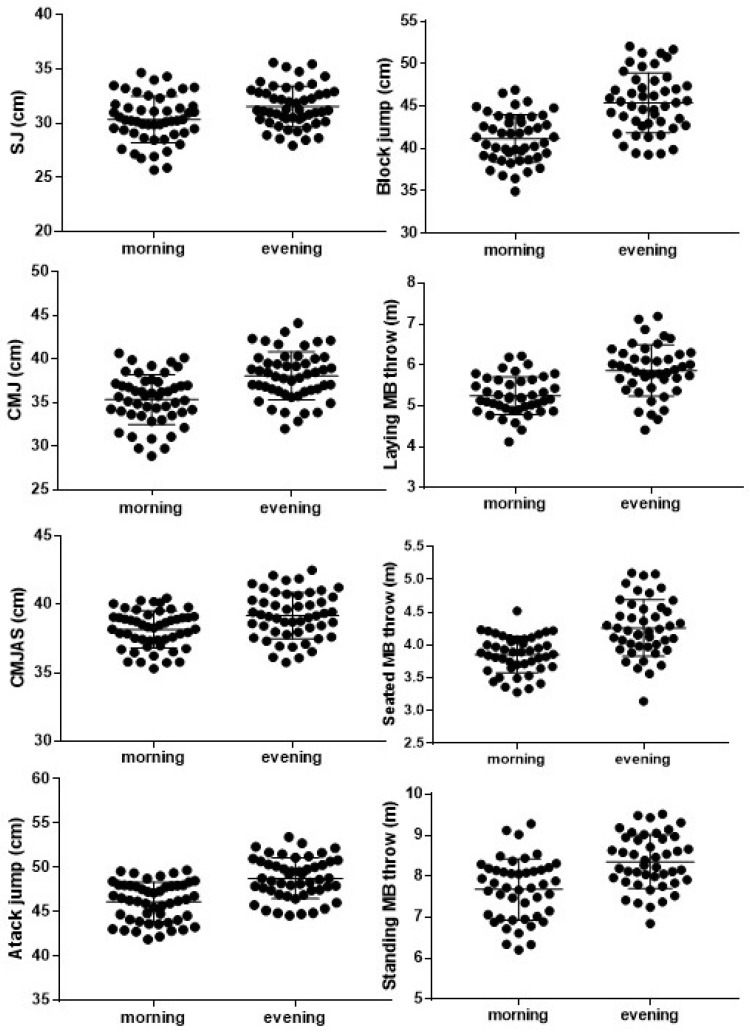
Individual differences between morning and evening testing.

Means ± SD for each upper and lower body power test measured in the morning and evening are shown in Table 2. Additionally, we observed that most performance tests did not exhibit substantial individual variability between morning and evening sessions, indicating consistent responses across participants. Results showed only notable variability primarily in upper body power measures (Figure 1). Significant differences (*p* < 0.05) in vertical jump were observed when comparing the morning and evening performance except for CMJAS, where there were no significant differences (*p* = 0.21). Specifically, in the SJ test, the jump height in the evening was significantly higher than in the morning, with an average increase of 1.5 cm (morning: 30.01 ± 4.36 cm; evening: 31.53 ± 4.21 cm, *p* = 0.05). Similarly, for the CMJ without arm swing, the evening values were higher by 2.4 cm (morning: 35.26 ± 5.55 cm; evening: 37.62 ± 5.68 cm, *p* = 0.02). Significant differences were also observed in the attack jump (morning: 45.66 ± 4.48 cm; evening: 47.85 ± 6.16 cm, *p* = 0.02) and block jump (morning: 42.36 ± 5.08 cm; evening: 44.42 ± 6.06 cm, *p* = 0.04).

The results for the upper body power tests revealed a significant difference only in the standing MB throw between morning and evening performances (Table 2). Athletes showed a greater throw distance in the evening, with an average increase of 0.6 m (morning: 7.70 ± 1.67 m; evening: 8.29 ± 1.73 m, *p* = 0.05; Cohen’s d = 0.35). However, no significant difference was observed in the lying MB throw (morning: 5.54 ± 1.18 m; evening: 5.86 ± 1.28 m, *p* = 0.75) and seated MB throw (morning: 3.92 ± 0.70 m; evening: 4.03 ± 0.91 m, *p* = 0.45).

## 4. Discussion

This study investigated morning to evening variations in upper and lower body power performance among young volleyball players. The biggest differences were found in lower body power performance between morning and evening testing. The results showed that vertical jump was much better in the evening compared to morning. On the contrary, we found no differences in upper body power performance except for standing medicine ball throw.

Investigating the differences between morning and evening power testing in volleyball players, particularly younger ones, is crucial for optimizing both training and performance evaluation. Younger athletes are still undergoing physiological development, which may make them more susceptible to circadian variations in strength and power output. Identifying the best times for performance testing can ensure that assessments are accurate and reflective of their true abilities. Moreover, this understanding can guide coaches in tailoring training schedules to maximize performance, reduce fatigue, and prevent injury in young volleyball players.

Morning-to-evening differences in performance in team sport athletes can be attributed to several physiological and hormonal factors. Circadian rhythms, which regulate body temperature, are often at their lowest in the morning, affecting muscle function and reducing peak strength and power [11]. Hormonal levels, such as testosterone, which supports muscle repair and performance, typically increase later in the day [19]. Additionally, cognitive functions such as reaction time improve in the afternoon, enhancing athletic performance [34,35].

The highest magnitude of change in power testing was observed for vertical jump tests. Leg power may be better in the evening due to several physiological factors related to circadian rhythms. Body temperature, which typically peaks later in the day, plays a key role in muscle flexibility and contraction efficiency, leading to better explosive power in the evening [11,25]. Additionally, the levels of key hormones like cortisol and testosterone, which influence muscle strength and recovery, tend to be more favorable for physical performance during the afternoon and evening [19,26]. This results in enhanced neuromuscular coordination and greater leg power output.

Specifically, better performance of jumps in volleyball during the evening compared to the morning can be attributed to physiological and chronobiological factors. According to Pivovarnicek et al. [7], athletes’ circadian preferences align more favorably with evening sessions, enhancing physical performance. Zhu and Cui [16] noted that plyometric jump training is more effective later in the day due to increased neuromuscular activation. Martín-López et al. [17] found that evening sessions optimize muscle coordination and power, leading to improved jumping ability in volleyball players due to heightened neuromuscular efficiency.

Contrary to the leg power, there were no significant differences in upper body power, except for standing medicine throw. The differences in leg power, but not upper body power, between evening and morning testing in volleyball could be due to the fact that leg muscles are more influenced by diurnal variations in body temperature and neuromuscular coordination. Lower body exercises like jumping are highly dependent on explosive power and muscle activation, which improve with increased body temperature and hormone levels later in the day. In contrast, upper body movements may rely more on muscular endurance and control, which are less affected by these circadian fluctuations. Regarding the upper body assessments, the standing medicine ball throw appears more reflective of volleyball-specific movements, in contrast to the lying and sitting medicine ball throws, which may be less applicable. The lack of significant differences in the lying and medicine ball throws suggests that additional or different upper body tests, more specific to volleyball actions, may be necessary to better assess these performance aspects.

Differences in morning and evening performance were observed across various team sports. Mhenni et al. [15] found that female handball players exhibit better short-term maximal physical performance in the evening, while Pavlović et al. [6] noted similar trends in elite male handball players. Essid et al. [3] highlighted better physical and psychological responses in young handball players later in the day. Basketball players also perform better in the afternoon [4], and young soccer players show improved performance in evening sessions [5]. These differences suggest the importance of time-of-day in athletic performance optimization.

While the hypothesis that both lower and upper body strength would improve in the evening was supported for lower body power, the effects on upper body performance were less consistent. The evening advantage for lower body explosive movements aligns with previous research, while the minimal impact on upper body power may suggest that the time-of-day effect is more pronounced for certain physical capacities. This finding highlights the need for further research to better understand the specific conditions under which these timing effects occur. Nevertheless, the current findings have practical implications for coaches and sports professionals, particularly in optimizing training schedules for adolescent athletes. Since lower body power was significantly better in the evening, coaches might consider scheduling training sessions focused on leg power movements, such as sprints, jumps, and other plyometric exercises, later in the day to maximize performance. On the other hand, the minimal time-of-day effect on upper body power suggests that upper body workouts, such as weightlifting or resistance training, may be less sensitive to timing, allowing for more flexibility in scheduling these sessions throughout the day. These insights can help coaches design more effective training plans by aligning the timing of exercises with the body’s natural performance peaks, potentially enhancing both performance and overall training outcomes.

However, some limitations should be mentioned. Factors such as individual circadian preferences and sleep patterns were not controlled, which could influence performance outcomes. Variability in testing conditions, such as differences in motivation or nutrition, may also affect the results. Although a sample size of 50 male adolescents showed sufficient statistical power, a larger sample size, including a more diverse population, would enhance the robustness of the findings and provide a more comprehensive understanding of the time-of-day effects on performance. Furthermore, the study’s findings are based on a specific group of adolescent male participants, which limits the generalizability to other populations, such as female athletes or individuals of different age groups. Future research should aim to include a more diverse sample to explore how these time-of-day effects might vary across different demographic groups and sports. A potential limitation of this study is the adolescent age of the participants, as maturity and growth rates can significantly influence performance outcomes. Adolescents experience rapid physical and physiological changes during this period, which may lead to varying responses in strength and power across different times of the day [36]. These developmental factors, such as muscle mass, hormonal fluctuations, and neuromuscular coordination, could introduce variability into the results and should be considered when interpreting the morning-to-evening performance differences in both lower and upper body power. While the tests used in this study were valid and reliable, they primarily assessed power using field-based measures. To improve the accuracy of strength assessments, especially for upper body strength, future studies should incorporate dynamometers to quantify strength more precisely. Lastly, the study may not account for long-term adaptations to morning or evening training, limiting the generalizability of the findings to short-term testing environments.

## 5. Conclusions

The study revealed that lower body power, as measured by vertical jumps, was significantly higher in the evening compared to the morning. In contrast, for upper body power, only the standing medicine ball throw showed significant differences between morning and evening testing, likely due to the similarity between the standing medicine ball throw and certain volleyball movements. These findings lead to several practical recommendations, especially for volleyball coaches. First, testing protocols should be scheduled in the evening for assessments of lower body power to capture peak performance levels. For upper body assessments, the standing medicine ball throw is recommended, as it appears more reflective of volleyball-specific movements, while the lying and sitting throws may be less relevant. Coaches might also consider scheduling lower body-focused training later in the day to align with athletes’ natural performance peaks. Practical guidelines for training timing include structuring evening sessions around lower body power exercises, while upper body training may have more flexibility in timing due to less variation across the day. Future research could explore broader age ranges and female athletes to assess if these diurnal variations are consistent across different groups.

## Figures and Tables

**Table 1:** Descriptive characteristics of participants.

	AS ± SD
Age (years)	14.5 ± 0.5
Body height (cm)	181.97 ± 5.29
Body mass (kg)	74.45 ± 8.61
Training background (years)	4.8 ± 0.9

**Table 2:** Upper and lower body power test results in the morning and evening.

	Morning (Mean ± SD)	Evening (Mean ± SD)	*p* Value	ES	CI	Magnitude
SJ (cm)	30.01 ± 4.36	31.53 ± 4.21	0.05	0.35	0.01 to 0.72	small increase
CMJ (cm)	35.26 ± 5.55	37.62 ± 5.68	0.02	0.42	0.06 to 0.78	small increase
CMJAS (cm)	38.28 ± 4.06	39.34 ± 5.21	0.21	0.23	−0.13 to 0.59	small increase
Attack jump (cm)	45.66 ± 4.48	47.85 ± 6.16	0.02	0.41	0.05 to 0.77	small increase
Block jump (cm)	42.36 ± 5.08	44.42 ± 6.06	0.04	0.37	0.01 to 0.73	small increase
Laying MB throw (m)	5.54 ± 1.18	5.86 ± 1.28	0.75	0.26	−0.10 to 0.62	small increase
Seated MB throw (m)	3.92 ± 0.70	4.03 ± 0.91	0.45	0.14	−0.22 to 0.49	trivial
Standing MB throw (m)	7.70 ± 1.67	8.29 ± 1.73	0.05	0.35	−0.01 to 0.71	small increase

*p* < 0.05 indicate statistical significance; ES—Effect Size; CI—Confidence intervals; SJ—squat jump; CMJ—counter movement jump; CMJAS—counter movement jump with arm swing; MB—medicine ball; Magnitude—effect size interpretation.

## Data Availability

The data presented in this study are available from the corresponding author upon request.

## References

[1] Lidor R., Ziv G. (2010). Physical characteristics and physiological attributes of adolescent volleyball players—A Review. Pediatr. Exerc. Sci..

[2] Malina R.M., Rogol A.D., Cumming S.P., e Silva M.J., Figueiredo A.J. (2015). Biological maturation of youth athletes: Assessment and implications. Br. J. Sports Med..

[3] Essid S., Cherif M., Chtourou H., Souissi N. (2022). Time-of-day effects in physical performances and psychological responses in young elite male handball players. Biol. Rhythm Res..

[4] Heishman A.D., Curtis M.A., Saliba E.N., Hornett R.J., Malin S.K., Weltman A.L. (2017). Comparing performance during morning vs. afternoon training sessions in intercollegiate basketball players. J. Strength Cond. Res..

[5] Chtourou H., Hammouda O., Souissi H., Chamari K., Chaouachi A., Souissi N. (2012). Diurnal variations in physical performances related to football in young soccer players. Asian J. Sports Med..

[6] Pavlović L., Stojiljković N., Aksović N., Stojanović E., Valdevit Z., Scanlan A.T., Milanović Z. (2018). Diurnal Variations in Physical Performance: Are there Morning-to-Evening Differences in Elite Male Handball Players?. J. Hum. Kinet..

[7] Pivovarnicek P., Popelka J., Plaskur D.T., Kentiba E., Hank M. (2023). Relationships between circadian preference and diurnal training times of Slovak Men’s Volleyball League. Biol. Rhythm Res..

[8] Roveda E., Mulè A., Galasso L., Castelli L., Scurati R., Michielon G., Esposito F., Caumo A., Montaruli A. (2020). Effect of chronotype on motor skills specific to soccer in adolescent players. Chronobiol. Int..

[9] Refinetti R. (2011). Integration of Biological Clocks and Rhythms. Compr. Physiol..

[10] Chtourou H., Souissi N. (2012). The effect of training at a specific time of day: A review. J. Strength Cond. Res..

[11] Drust B., Waterhouse J., Atkinson G., Edwards B., Reilly T. (2005). Circadian rhythms in sports performance—An update. Chronobiol. Int..

[12] Teo W., Newton M.J., McGuigan M.R. (2011). Circadian Rhythms in Exercise Performance: Implications for Hormonal and Muscular Adaptation. J. Sports Sci. Med..

[13] Sedliak M., Finni T., Peltonen J., Häkkinen K. (2008). Effect of Time-of-Day-Specific Strength Training on Maximum Strength and EMG Activity of the Leg Extensors in Men. J. Sports Sci..

[14] Reilly T., Waterhouse J. (2009). Circadian Aspects of Body Temperature Regulation in Exercise. J. Therm. Biol..

[15] Mhenni T., Michalsik L.B., Mejri M.A., Yousfi N., Chaouachi A., Souissi N., Chamari K. (2017). Morning–evening difference of team-handball-related short-term maximal physical performances in female team handball players. J. Sports Sci..

[16] Zhu M., Cui Z. (2024). Chronobiological Insights in Plyometric Jump Training: Optimizing Sport-Performance Adaptations for Volleyball Players. Int. J. Sports Physiol. Perform..

[17] Martín-López J., Sedliak M., Valadés D., Muñoz A., Buffet-García J., García-Oviedo R., Rodríguez-Aragón M., Pérez-López A., López-Samanes Á. (2022). Impact of time-of-day and chronotype on neuromuscular performance in semi-professional female volleyball players. Chronobiol. Int..

[18] Lloyd R.S., Oliver J.L. (2012). The Youth Physical Development Model: A New Approach to Long-Term Athletic Development. Strength Cond. J..

[19] Ayala V., Martínez-Bebia M., Latorre J.A., Gimenez-Blasi N., Jimenez-Casquet M.J., Conde-Pipo J., Bach-Faig A., Mariscal-Arcas M. (2021). Influence of circadian rhythms on sports performance. Chronobiol. Int..

[20] Knaier R., Qian J., Roth R., Infanger D., Notter T., Wang W., Scheer F.A. (2022). Diurnal Variation in Maximum Endurance and Maximum Strength Performance: A Systematic Review and Meta-Analysis. Med. Sci. Sports Exerc..

[21] Oueslati G., Ouergui I., Ammar A., Trabelsi K., Ardigò L.P., Chtourou H. (2024). Diurnal Variation of Psychomotor, Cognitive, and Physical Performances in Schoolchildren: Sex Comparison. BMC Pediatr..

[22] Mirwald R.L., Baxter-Jones A.D., Bailey D.A., Beunen G.P. (2002). An assessment of maturity from anthropometric measurements. Med. Sci. Sports Exerc..

[23] Jones M.A., Hitchen P.J., Stratton G. (2000). The importance of considering biological maturity when assessing physical fitness measures in girls and boys aged 10 to 16 years. Ann. Hum. Biol..

[24] Sattler T., Hadžic V., Derviševic E., Markovic G. (2015). Vertical jump performance of professional male and female volleyball players: Effects of playing position and competition level. J. Strength Cond. Res..

[25] Sattler T., Sekulic D., Hadzic V., Uljevic O., Dervisevic E. (2012). Vertical jumping tests in volleyball: Reliability, validity and playing-position specifics. J. Strength Cond. Res..

[26] Marelić N., Đurković T., Rešetar T. (2008). Razlike u kondicijskim sposobnostima i morfološkim karakteristikama odbojkašica različitog statusa u ekipi. Croat. Sports Med. J./Hrvatski Sportskomed. Vjesn..

[27] Nejić D., Trajković N., Stanković R., Milanović Z., Sporiš G. (2013). A comparison of the jumping performance of female junior volleyball players in terms of their playing positions. Facta Univ. Ser. Phys. Educ. Sport.

[28] Sheppard J.M., Cronin J.B., Gabbett T.J., McGuigan M.R., Etxebarria N., Newton R.U. (2008). Relative importance of strength, power, and anthropometric measures to jump performance of elite volleyball players. J. Strength Cond. Res..

[29] Tomljanovic M., Spasic M., Gabrilo G., Uljevic O., Foretic N. (2011). Effects of five weeks of functional vs. traditional resistance training on anthropometric and motor performance variables. Kinesiology.

[30] Trajković N., Madić D., Andrašić S., Milanović Z., Radanović D. (2017). Effects of medicine ball training on physical fitness in primary school children. Facta Univ. Ser. Phys. Educ. Sport.

[31] Marques M.C., Van den Tillaar R., Gabbett T.J., Reis V.M., González-Badillo J.J. (2009). Physical fitness qualities of professional volleyball players: Determination of positional differences. J. Strength Cond. Res..

[32] Van Den Tillaar R., Marques M.C. (2013). Effect of Different Training Workload on Overhead Throwing Performance with Different Weighted Balls. J. Strength Cond. Res..

[33] Hopkins W., Marshall S., Batterham A., Hanin J. (2009). Progressive statistics for studies in sports medicine and exercise science. Med. Sci. Sports Exerc..

[34] Nobari H., Azarian S., Saedmocheshi S., Valdés-Badilla P., Calvo T.G. (2023). Narrative review: The role of circadian rhythm on sports performance, hormonal regulation, immune system function, and injury prevention in athletes. Heliyon.

[35] Simmons N., Mandal S., Paton B., Ahmed I. (2022). Are Circadian Rhythms a New Frontier in Athletic Performance?. Curr. Sports Med. Rep..

[36] Armstrong N., McManus A.M. (2017). Physiology of elite young male athletes. Med. Sport Sci..

